# Cross-biome comparison of microbial association networks

**DOI:** 10.3389/fmicb.2015.01200

**Published:** 2015-10-27

**Authors:** Karoline Faust, Gipsi Lima-Mendez, Jean-Sébastien Lerat, Jarupon F. Sathirapongsasuti, Rob Knight, Curtis Huttenhower, Tom Lenaerts, Jeroen Raes

**Affiliations:** ^1^Center for the Biology of Disease, VIBLeuven, Belgium; ^2^Department of Microbiology and Immunology, REGA Institute, KU LeuvenLeuven, Belgium; ^3^Department of Applied Biological Sciences, Vrije Universiteit BrusselBrussels, Belgium; ^4^Machine Learning Group, Department of Computer Science, Université Libre de BruxellesBrussels, Belgium; ^5^23andMe Inc., Mountain ViewCA, USA; ^6^Department of Chemistry and Biochemistry and BioFrontiers Institute, University of Colorado, BoulderCO, USA; ^7^Department of Biostatistics, Harvard School of Public Health, BostonMA, USA; ^8^Artificial Intelligence Lab, Department of Computer Science, Vrije Universiteit BrusselBrussels, Belgium; ^9^Interuniversity Institute of Bioinformatics in Brussels, Université Libre de Bruxelles–Vrije Universiteit BrusselBrussels, Belgium

**Keywords:** microbial communities, 16S rDNA sequencing, co-occurrence, network comparison, positive edge percentage, evenness

## Abstract

Clinical and environmental meta-omics studies are accumulating an ever-growing amount of microbial abundance data over a wide range of ecosystems. With a sufficiently large sample number, these microbial communities can be explored by constructing and analyzing co-occurrence networks, which detect taxon associations from abundance data and can give insights into community structure. Here, we investigate how co-occurrence networks differ across biomes and which other factors influence their properties. For this, we inferred microbial association networks from 20 different 16S rDNA sequencing data sets and observed that soil microbial networks harbor proportionally fewer positive associations and are less densely interconnected than host-associated networks. After excluding sample number, sequencing depth and beta-diversity as possible drivers, we found a negative correlation between community evenness and positive edge percentage. This correlation likely results from a skewed distribution of negative interactions, which take place preferentially between less prevalent taxa. Overall, our results suggest an under-appreciated role of evenness in shaping microbial association networks.

## Introduction

Microorganisms engage in a multitude of ecological interactions, ranging from mutualism to parasitism and competition (Konopka, 2009). These interactions shape species distributions, and should thus be detectable from co-occurrence patterns across different locations, replicates or time points (Diamond, 1975; Horner-Devine et al., 2007; Hekstra and Leibler, 2012).

Network inference techniques are increasingly employed to decipher microbial relationships from such patterns (reviewed in Faust and Raes, 2012). These techniques include simple pair-wise Pearson or Spearman correlations (Arumugam et al., 2011; Barberán et al., 2012; in Zhou et al., 2010, coupled with random matrix theory), local similarity analysis (LSA; Ruan et al., 2006; Xia et al., 2011, 2013; Durno et al., 2013), compositionality-robust estimation of correlations (SparCC; Friedman and Alm, 2012, REBACCA; Ban et al., 2015, CCLasso; Fang et al., 2015), Gaussian graphical models (Van den Bergh et al., 2012; Kurtz et al., 2015), sparse regression (Faust et al., 2012), and assessment of co-occurrence probability with the hypergeometric distribution for presence/absence data (Chaffron et al., 2010; Freilich et al., 2010). In food webs, Bayesian regression is also applied (Faisal et al., 2010; Aderhold et al., 2012).

Previously, we developed a pipeline based on an ensemble approach (Faust et al., 2012), which we used recently to predict interactions in the oceanic plankton community (Lima-Mendez et al., 2015). This pipeline combines a number of measures of dependency, such as correlation (e.g. Spearman), similarity (e.g. mutual information), and dissimilarity (e.g. Kullback–Leibler). The rationale behind this ensemble approach is that different measures make different errors, but tend to agree on the correct associations. This “wisdom of crowds” metaheuristic approach has been demonstrated to deliver robust and accurate results for gene regulatory networks (Marbach et al., 2012).

To remove spurious correlations that stem from differences in sequencing depth, samples need to be rarefied or normalized, which constrains the total sample count and thus introduces compositionality bias (Aitchison, 2003). To address this bias, we include the Bray–Curtis and Kullback–Leibler dissimilarities, which are not affected by it, and apply the ReBoot procedure to correlation measures, which mitigates compositionality bias (Faust et al., 2012).

We then studied whether and how microbial association networks differ across biomes. Microbial community composition and diversity (e.g. Lozupone and Knight, 2007; Fierer and Lennon, 2011) as well as properties of co-occurrence networks have been compared previously. Microbial network properties considered in comparisons include among others the number of edges as a measure of complexity (Dini-Andreote et al., 2014), the network diameter, density, average path length, and clustering coefficient (Peura et al., 2015) and the module number (Williams et al., 2014). In some cases, interesting ecological insights can be gained from a network comparison. For instance, the extent of network fragmentation after node deletion has been applied as a measure of robustness to random or targeted species removal (Widder et al., 2014; Peura et al., 2015) as well as a measure of stochasticity (Widder et al., 2014). Widder and co-workers found a lower network fragmentation for river regions with intermediate catchment areas as compared to those with large or small catchment areas. They explain this observation by a stronger hydrological variability and higher dispersal limitation upstream and a larger number of source communities down-stream as two different sources of increased stochasticity in these river regions (Widder et al., 2014). Furthermore, the consistency of individual taxon links can be evaluated by cross-network comparison (Williams et al., 2014; Xu et al., 2014). The effect of various network properties on co-occurrence network inference accuracy has also been intensively studied (Berry and Widder, 2014).

However, the potential impact of community properties such as alpha and beta diversity on the properties of co-occurrence networks has not yet been well explored, though it is crucial for the interpretation of these network properties. In addition, previous network studies mostly focus on a single biome. We therefore built 20 biome-specific networks from 7 environmental and 13 host-associated sample sets, which together span 11 biomes and which differ widely in their sample and taxon number as well as their sequencing depth and community properties. We then examined whether these factors affected network properties.

## Materials and Methods

### Data Acquisition and Preprocessing

The QIIME database (now a part of Qiita; The Qiita Development Team, 2015) provides sequence data uniformly processed with the QIIME pipeline (Caporaso et al., 2010) as well as sample metadata in a standardized format, supporting MIMARKS (minimum information about a marker gene sequence; Yilmaz et al., 2011). Operational taxonomic units (OTUs) are clustered at 97% identity using UCLUST-ref (Edgar, 2010) against the Greengenes 16S rRNA gene database (DeSantis et al., 2006). Samples are classified using the environment ontology^1^ and anatomy ontology (UBERON; Mungall et al., 2012), respectively, which allows stratifying QIIME data according to biome and body area. We filtered the global OTU count matrix obtained from the QIIME database in July 10th, 2011 to discard OTUs or metadata with less than 50 occurrences across all samples. Soil-biome specific OTU matrices were then obtained by filtering on the BIOME_ENVO terms. The tundra biome is composed of all “Tundra communities and barren Arctic deserts” samples, the moist forest biome of all “Tropical and subtropical moist broadleaf forest biome” samples, the coniferous forest biome of all “Tropical and subtropical coniferous forest biome” samples and the grassland biome of all “Temperate grasslands, savannas, and shrubland biome” samples. The following UBERON terms were merged for the intestine biome: “cecum,” “colon,” “stomach,” “small intestine,” “large intestine,” “rectum,” and “feces.” In case of the skin biome, the following UBERON terms were selected: “skin,” “skin of arm,” “skin of digit of hand,” “skin of finger,” “skin of forearm,” “skin of head,” “zone of skin of head,” “zone of skin of hand,” “zone of skin of knee,” “zone of skin of outer ear,” “zone of skin of abdomen,” “zone of skin of foot,” “zone of skin of wrist,” “nose,” “fossa,” and “glans penis.” Oral cavity terms included “mouth,” “mucosa of mouth,” “tongue,” “buccal mucosa,” “gingiva,” “gingival epithelium,” “hard palate,” “mucosa of tongue,” “oral cavity,” “oropharynx,” and “palatine tonsil.”

Each biome-specific count matrix was then processed as follows: All OTUs that occurred in less than 1/4th of its samples and all samples with a sequencing depth (i.e. a total number of reads) within the lower 25% of its sequencing depth range were discarded in this order. Counts were then converted into relative abundances by dividing each entry by the sum of its corresponding sample. To explore potential associations at higher taxonomic ranks, the taxa composing the OTU lineages were added as additional entries to the matrices. Higher-level taxon abundances were then obtained as the sum of member OTU relative abundances. During all steps of network construction, links between taxa with a parent–child relationship (e.g. between *Escherichia* and Enterobacteriaceae) were forbidden. It is of note that higher-level taxa with a single member only form the same associations as their member taxon. Super-kingdom taxa (Bacteria, Archaea) are not considered.

To compare networks constructed from relative abundances and from rarefied counts, we rarefied count data to 600 counts per sample, which resulted in the loss of 86 QIIME soil samples (41 for coniferous forest, 17 for grasslands, 25 for moist forest, and 3 for tundra).

Human Microbiome Project (HMP) 16S V35 data (Methé et al., 2012) were downloaded from the QIIME database in December 2012 in biom format, converted with the biom convert tool (McDonald et al., 2012) and processed as described above. In addition, samples flagged as mislabeled or contaminated in the metadata were removed. In addition to intestine, oral cavity and skin matrices, vagina (with terms: “labia minora,” “mucosa of vagina,” “vaginal fornix,” “vagina”) and nasal cavity (“nasal cavity,” “nostrils,” “nostril,” “nares”) matrices were extracted. The samples of these body-area specific matrices were split by recruitment center (“11BAY” and “92WAU”) to address a known batch effect and the same filtering steps described above were carried out for each of these sample sets. Thus, 10 networks were constructed: two networks for each of the five body areas (intestine, oral cavity, nasal cavity, skin, and vagina).

Earth Microbiome Project (EMP) data (Gilbert et al., 2010) for the studies: Caporaso_Glen_Canyon_soils (ENVO term: “anthropogenic terrestrial biome”), Gittel_CryoCARB_2_permafrost (ENVO term: “tundra biome”) and Dubinsky_Hawaii_Kohala (ENVO term: “tropical shrubland biome”) were downloaded in January 2014 from the EMP database (now a part of Qiita) in biom format and count matrices were preprocessed in the same way as QIIME biome-specific count matrices. In total, 463 soil and 6,357 host samples were analyzed.

### Network Inference

For each of four similarity measures (Bray–Curtis and Kullback–Leibler dissimilarity, Pearson and Spearman correlation), a distribution of all pair-wise scores was computed. Given these distributions, initial thresholds were selected such that each measure contributed 1,000 positive and 1,000 negative edges to the initial network. For each measure and each edge, 1,000 renormalized permutation and bootstrap scores were generated, following the ReBoot routine, which alleviates compositionality bias (Faust et al., 2012). The measure-specific *p*-value was then computed as the probability of the null value (i.e. the mean of the null distribution) under a Gauss curve generated from the mean and standard deviation of the bootstrap distribution. Since a one-sided test was carried out, *p*-values above 0.5 were considered indicative of mutual exclusion and were converted by subtraction from one. Next, measure-specific *p*-values were merged using Brown’s (1975) method, which takes correlations among measures into account (i.e. an edge supported by two inversely correlated measures will receive a lower *p*-value than one supported by two correlated measures). After multiple-testing correction using Benjamini and Hochberg’s (1995) procedure, edges with merged *p*-values below 0.05 were kept. Any edge for which the four measures did not agree on the interaction type (i.e. positive or negative) or whose initial interaction type contradicted the interaction type determined by the *p*-value was also discarded. This network construction protocol is the same as the one applied in (Faust et al., 2012), but without the computationally intensive generalized boosted linear models, which clustered with the correlation measures (Faust et al., 2012) and with Brown’s method instead of Simes method, because Brown’s method takes dependencies among similarity score distributions into account. Network construction was carried out with CoNet^2^, which implements the pipeline described above. The Supplementary Material provides CoNet setting files as well as a bash script to re-run network inference within Cytoscape or on command line. The inferred networks are also available as a supplementary Cytoscape file. Networks in this study were constructed with CoNet alpha.

### Matrix and Network Property Calculation

Biome-specific matrices were rarefied to the same total count per sample (362) and higher-level taxa were not included for diversity calculation. Beta-diversity was then calculated as the median of all pair-wise Bray–Curtis scores computed sample-wise. The biome-specific Bray–Curtis distributions were visualized in a box plot (see Supplementary Figure S7). In addition, we computed the over-dispersion parameter θ of the Dirichlet–Multinomial distribution, which measures to which extent taxon abundances across samples will deviate from their average abundance (Rosa et al., 2012). We obtained biome-specific θ values by fitting a Dirichlet–Multinomial distribution to the count matrices using the dirmult R package (Tvedebrink, 2010). Alpha-diversity was calculated using the Shannon index, defined as 
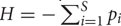
 ⋅ lnp_i_, where p_i_ is the proportion of species *i* and *S* is the species number. Chao1 (Chao, 1987), implemented in the R package vegan, was employed as richness estimator. Evenness is usually computed with the Pielou index (Pielou, 1975). However, this index is known to be influenced by species richness (Sheldon, 1969) and thus cannot be used to assess the impact of evenness independently of richness. Therefore, the corrected Sheldon index was chosen as evenness index (corresponding to formula *F*_1,0_ in (Alatalo, 1981)). The Pielou index is defined as J = H/lnS and the (corrected) Sheldon index as F = (N_1_ - 1)/(N_0_ - 1), where N_i_ = exp^H^ and N_0_ = S.

To quantify the connectedness of the networks, we computed the average clustering coefficient as the mean of all node-specific clustering coefficients. The node-specific clustering coefficient is defined as C_i_ = 2 ⋅ n/(k_i_ ⋅ (k_i_ - 1)) where *k_i_* is the number of neighbors of node *i* and *n* is the number of edges between the neighbors of node *i*, excluding node *i*. We also computed the average path length (which is the average length of all possible shortest paths in the network) and the network density (the ratio of realized to possible edge number). In addition, we quantified scale-freeness as the goodness of fit (using R2) of a power-law to the node degree distribution. Cluster coefficients were calculated with tYNA (Yip et al., 2006).

### Simulation Studies

Count matrices were generated from a Dirichlet–Multinomial distribution using the rmultinom function from the R stats package and the rdirichlet function from the MCMCpack package (Martin et al., 2011). Throughout all simulations, unless indicated otherwise, the over-dispersion parameter θ was set to 0.002, the total read number to 1,000 and each taxon probability to 1/S. The value for θ was chosen to lie within the range of θ values obtained for the biome-specific count matrices, which had θ values from 0.0009 (grasslands) to 0.34 (vaginal HMP). Networks were constructed from the count matrices by retaining all taxon pairs with Spearman correlations above 0.2 or below –0.2. In case *p*-values were computed, they were either obtained from a standard permutation test or by combining a permutation and a bootstrap distribution as described above, followed by Benjamini and Hochberg (1995) multiple-testing correction.

Count matrix evenness was varied by obtaining taxon probabilities from the geometric series for different values of the resource fraction parameter (May, 1975). Group structure was simulated by generating low background counts from a Dirichlet–Multinomial distribution with equal taxon probabilities while increasing the counts of selected taxa across a sub-set of samples.

The R code used to generate count matrices and to carry out network construction is provided as Supplementary Material.

## Results

### Biome-specific Networks Reproduce Known Associations and Predict Novel Ones

Uniformly processed 16S data sets were gathered from the QIIME database (Caporaso et al., 2010), the EMP database (Gilbert et al., 2010), and the HMP (Huttenhower et al., 2012; Methé et al., 2012). Biome-specific networks were constructed using four measures (Spearman, Pearson, Bray–Curtis, and Kullback–Leibler). Measure-specific as well as combined *p*-values were calculated for each edge that scored above an initial threshold and the final network was obtained by discarding edges with multiple-testing-corrected combined *p*-values above 0.05 (see Materials and Methods for details). Supplementary Tables S1 and S2 summarize the properties of the biome-specific input matrices and their resulting networks, respectively.

**FIGURE 1:**
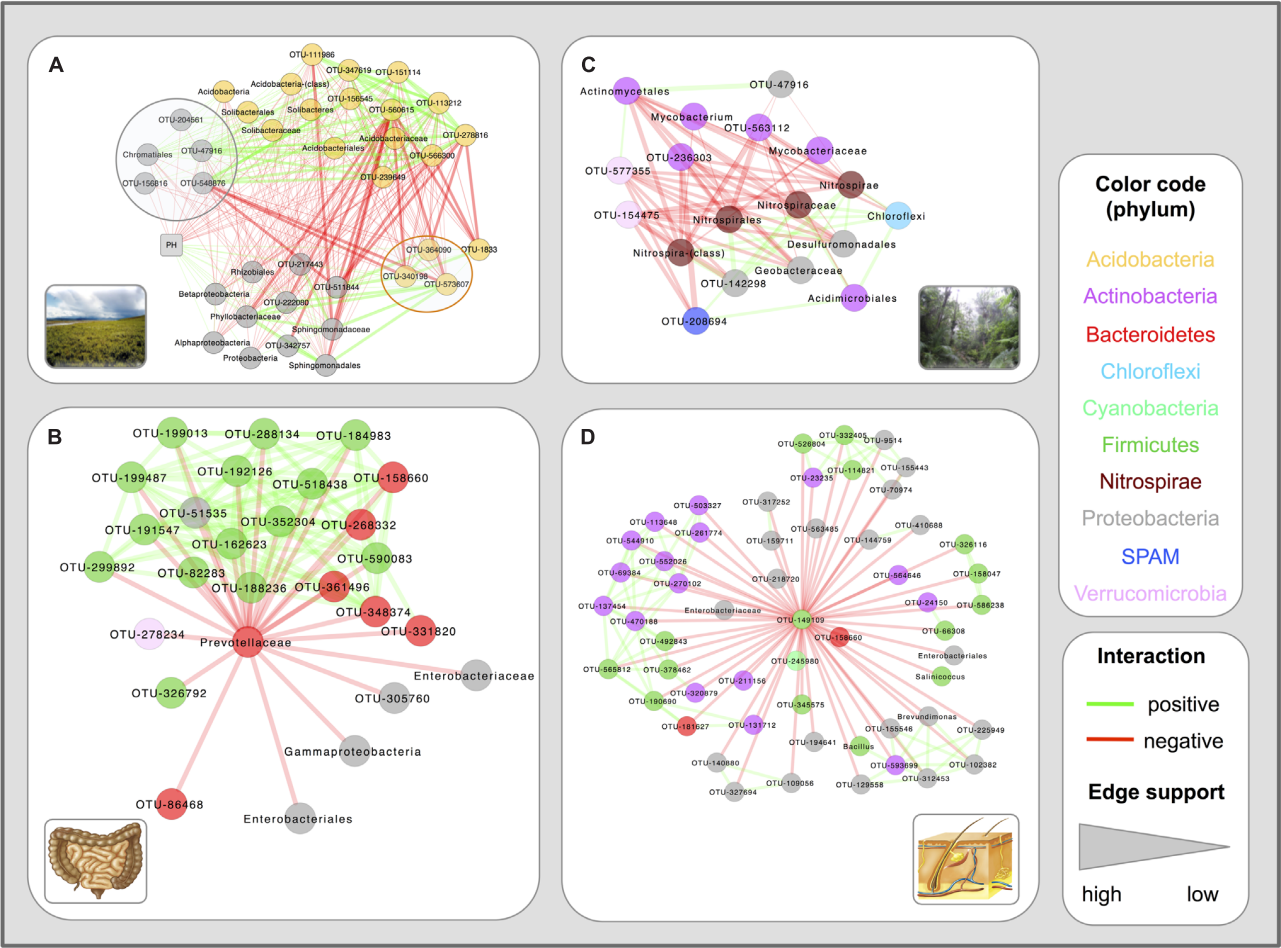
**Example networks constructed from QIIME 16S data.** Four sub-networks, i.e. node and edge sub-sets from the inferred networks, are shown. The tundra sub-network **(A)** is dominated by two mutually exclusive clusters consisting of Acidobacteria and Alphaproteobacteria, the first of which is anti-correlated and the second correlated to pH. Notable exceptions to this trend are the Chloracidobacteria (a class within the Acidobacteria, here highlighted with an orange circle) which are positively correlated to pH, and several Rhizobiales (Alphaproteobacteria) and Chromatiales (Gammaproteobacteria) members (gray circle), which are negatively correlated to pH. The gut sub-network **(B)** reproduces the *Prevotella* enterotype, including negative correlations of the Prevotellaceae to an *Akkermansia* and an *Escherichia* OTU as well as to several Bacteroides OTUs. The moist forest sub-network **(C)** displays the neighbors of higher-level Nitrospirae representatives, among them the Geobacteraceae, with which Nitrospirales members might cross-feed. The skin sub-network **(D)** shows the neighbors of a *Streptococcus* OTU that acts as a negative hub.

A closer inspection of the networks shows that several associations reproduce known microbial relationships. For example, the tundra network contains a node representing pH. Its neighbors form two mutually exclusive clusters: one positively correlated with pH, consisting mainly of members of the Alphaproteobacteria, and the other negatively correlated with pH, featuring mainly Acidobacteria (**Figure 1A**). However, OTUs of the Acidobacteria family Chloracidobacteria correlate positively with pH, whereas OTUs of the Rhizobiales order within the Alphaproteobacteria are inversely correlated to pH. The cluster structure thus allows a more fine-grained interpretation of the previously detected phylum- and class-level (anti-) correlations between pH and Acidobacteria and Alphaproteobacteria (Chu et al., 2010). Another example is the sub-network composed of the Prevotellaceae node and its neighbors in the gut network (**Figure 1B**), which reproduces the *Prevotella* enterotype reported in (Arumugam et al., 2011). For instance, it captures the negative relationships of Prevotellaceae to members of the *Akkermansia* and *Escherichia* genera and several OTUs of *Bacteroides* (the main driver of another enterotype), which are positively correlated among themselves and with a number of Firmicutes (such as *Blautia, Faecalibacterium*, and *Roseburia*). We have reported such inverse correlations between enterotype drivers previously (Faust et al., 2012). The *Prevotella* enterotype has been shown to be clinically relevant in (Scher et al., 2013).

Beyond confirming known relationships, network construction can suggest novel ones. For instance, the tropical moist forest network contains a positive association of Nitrospirales (nitrite oxidizers) with Geobacteraceae (organic compound oxidizers; **Figure 1C**). The predicted Nitrospirales–Geobacteraceae association might reflect a cross-feeding relationship, where Geobacteraceae species use nitrate generated by Nitrospirales members as electron acceptor. Although Geobacteraceae are better known as metal reducers, several Geobacteraceae species are able to grow on nitrate as the sole electron acceptor (Kashefi et al., 2003; Kashima and Regan, 2015). Since Geobacteraceae species are anaerob and several members of Nitrospirales aerob, their interaction may take place indirectly via nitrate diffusing into deeper soil layers. The absence of relationships between member OTUs of Geobacteraceae and Nitrospirales may hint at functional redundancy: if all members of the Nitrospirales and Geobacteraceae groups perform a certain function (nitrite oxidation versus nitrate reduction), then any member of the first group can cross-feed with any member of the second group. In consequence, the group counts co-vary, even if individual group members could be randomly distributed.

Another example can be found in the skin network, which is dominated by several hubs (that is highly connected nodes; an example is depicted in **Figure 1D**). The hub OTUs are all members of the *Streptococcus* and *Staphylococcus* genera, which are dominant members of the normal skin flora. Although the negative hubs may reflect an invasion of the normal skin microbiota by more aggressive (in some contexts pathogenic) species, an alternative interpretation is that they result from different responses of skin genera to hygiene: *Streptococcus* and *Staphylococcus* members are more abundant on recently washed hands, whereas other genera increase in abundance with time after hand washing (Fierer et al., 2008). Both interpretations may be related: early-colonizing genera may take advantage of a reduced skin microbiota, to be replaced by a more mature skin community later on. Such a link between early colonizers and pathogens has recently been suggested for gut species (Lozupone et al., 2012).

The vaginal HMP networks contain a *Lactobacillus* cluster and a mixed cluster composed of *Anaerococcus, Prevotella, Finegoldia, Peptoniphilus*, and other genera. *Lactobacillus iners* forms a negative hub in both networks. These associations are in agreement with the vaginal community types reported in (Ravel et al., 2011) and have been detected previously in HMP data (Faust et al., 2012; Friedman and Alm, 2012). In addition, the oral cavity HMP networks, which contain dental plaque samples, reproduce known relationships between early (*Streptococcus*), intermediate (*Fusobacterium*) and late colonizers (e.g. *Selenomonas, Tannerella, Treponema, Prevotella*) of the dental plaque (Kolenbrander et al., 2002). These associations have also been inferred previously from HMP data (Faust et al., 2012; Friedman and Alm, 2012).

### Soil and Host Networks Differ in their Network Properties

**FIGURE 2:**
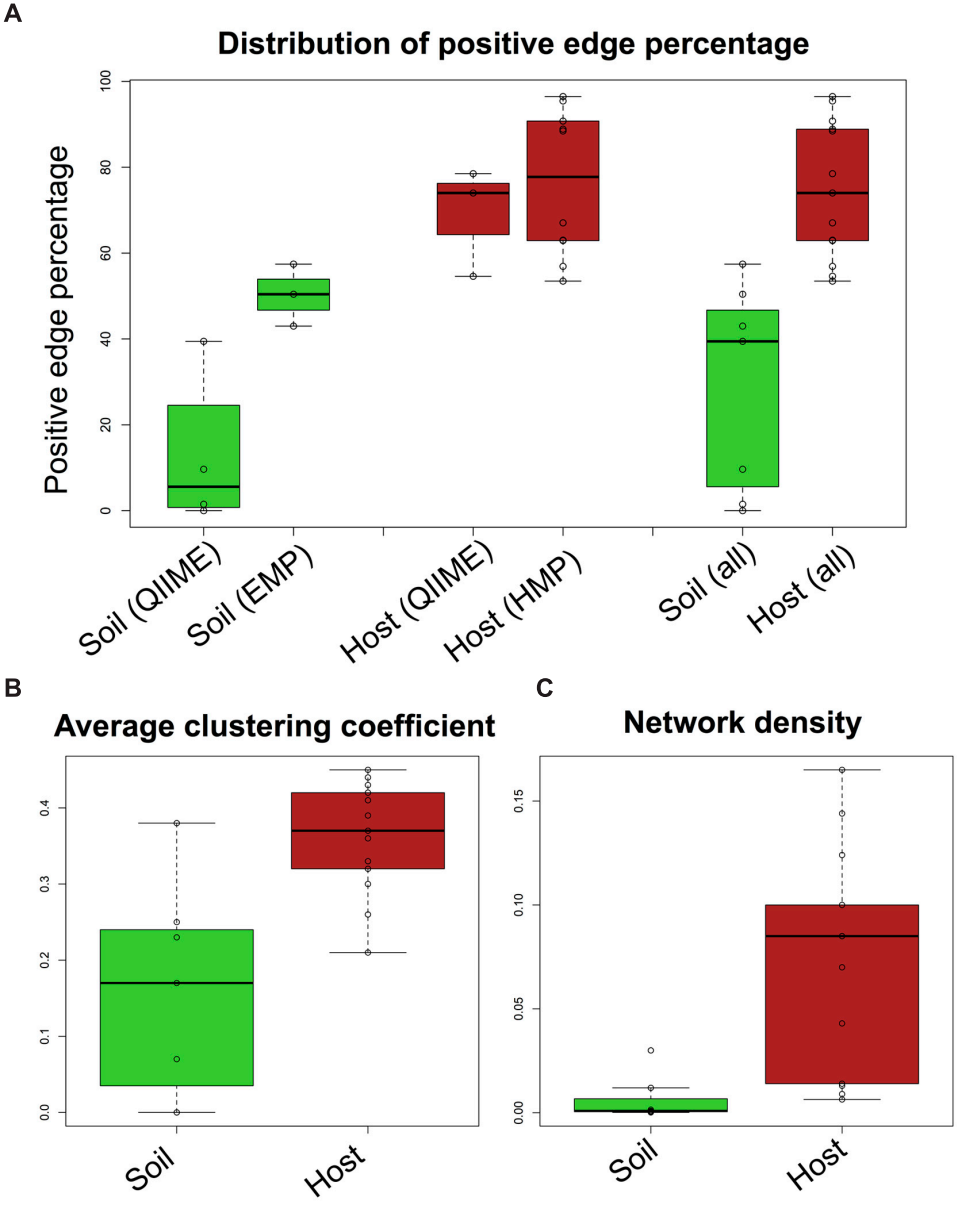
**Differences between host and soil networks.** Soil networks fall into two groups, characterized by low (QIIME soils) and high sequencing depth [Earth Microbiome Project (EMP) soils], whereas host networks constructed from QIIME and Human Microbiome Project (HMP) samples have comparable PEP. When taking all networks together, PEP in soil is significantly lower (*p*-value: 0.0002 according to the Wilcoxon rank sum test) than in host **(A)**. The average clustering coefficient **(B)** and network density **(C)** are also significantly different (*p*-values: 0.004 and 0.002, Wilcoxon rank sum test). Network density is computed as 2E/N(N-1), where E is the edge number and N the number of taxa in the processed matrix.

Comparing the properties of the calculated networks, we observed that host-associated networks contain a significantly higher percentage of positive edges (PEP, computed as the percentage of positive edges out of all realized edges) than soil networks according to Wilcoxon’s rank sum test (**Figure 2A**). The difference in PEP was accompanied by a significantly higher average clustering coefficient and network density in host networks as compared to soil networks (**Figures 2B,C**). We then constructed sub-networks consisting only of positive and of negative edges, respectively. Sub-networks consisting of only negative edges were found to have far lower average clustering coefficients than both the positive sub-networks and the full networks (Supplementary Figure S1A), suggesting that neighbors of a node tend to be interconnected by positive links. As expected, the positive and negative network densities reflect the proportion of positive and negative edges in the full networks (Supplementary Figure S1B). Given the dependency of the average clustering coefficient and network density on PEP (Spearman’s rho: 0.72 and 0.62, respectively), we focused on this latter property.

The question is whether the observed difference in PEP is due to a true biological process or caused by differences in sample processing or network construction biases. Previous work has shown that sequencing platform, DNA extraction protocol and amplified 16S rRNA variable region can partly drive the clustering of gut samples (Lozupone et al., 2013), whereas more recent work has highlighted the strong impact of sequencing depth (Weiss et al., 2015). The high PEP of host networks was reproduced with data sets sequenced with different platforms and 16S regions, whereas the soil PEP was more heterogeneous; its standard deviation was larger than in host (24% in soil versus 16% in host). EMP soils, which were sequenced with another platform and 16S region than QIIME soils, had a higher average PEP than QIIME soils, which was, however, still below the average PEP of the host networks (**Figure 2A**).

The host and soil datasets differ considerably in their sample number (averaging to 489 versus 66 samples per biome). To test the impact of sample number, we constructed networks from randomly selected sample subsets of the oral cavity QIIME and tropical shrubland EMP data and plotted the PEP distribution for each sample subset size (Supplementary Figure S2). The PEPs of these networks averaged to a value close to that computed for the full sample set, showing that sample number does not affect PEP.

Data sets also differ in their nature (time-series versus cross-sectional studies). While some sites were sampled once (tundra) or five times (NEON study) per year, many of the gut samples come from time series studies, (e.g. Turnbaugh et al., 2008; Caporaso et al., 2011) and the oral cavity samples all belong to a single longitudinal study (Caporaso et al., 2011). Fecal samples from the same person at different time points were found to be less heterogeneous than samples from different persons (Turnbaugh et al., 2008), pointing to a possible bias due to different proportions of time series.

To address whether the higher percentage of time series among host samples might contribute to the observed PEP difference, we constructed networks from time-series free sample sub-sets of skin (Fierer et al., 2008) and gut (Turnbaugh et al., 2008). The PEP of these networks did not differ substantially from their unfiltered counter-parts (78.5% versus 73.5% in skin and 54.6% versus 61.5% in gut).

### Sequencing Depth Impacts Positive Edge Percentage

**FIGURE 3:**
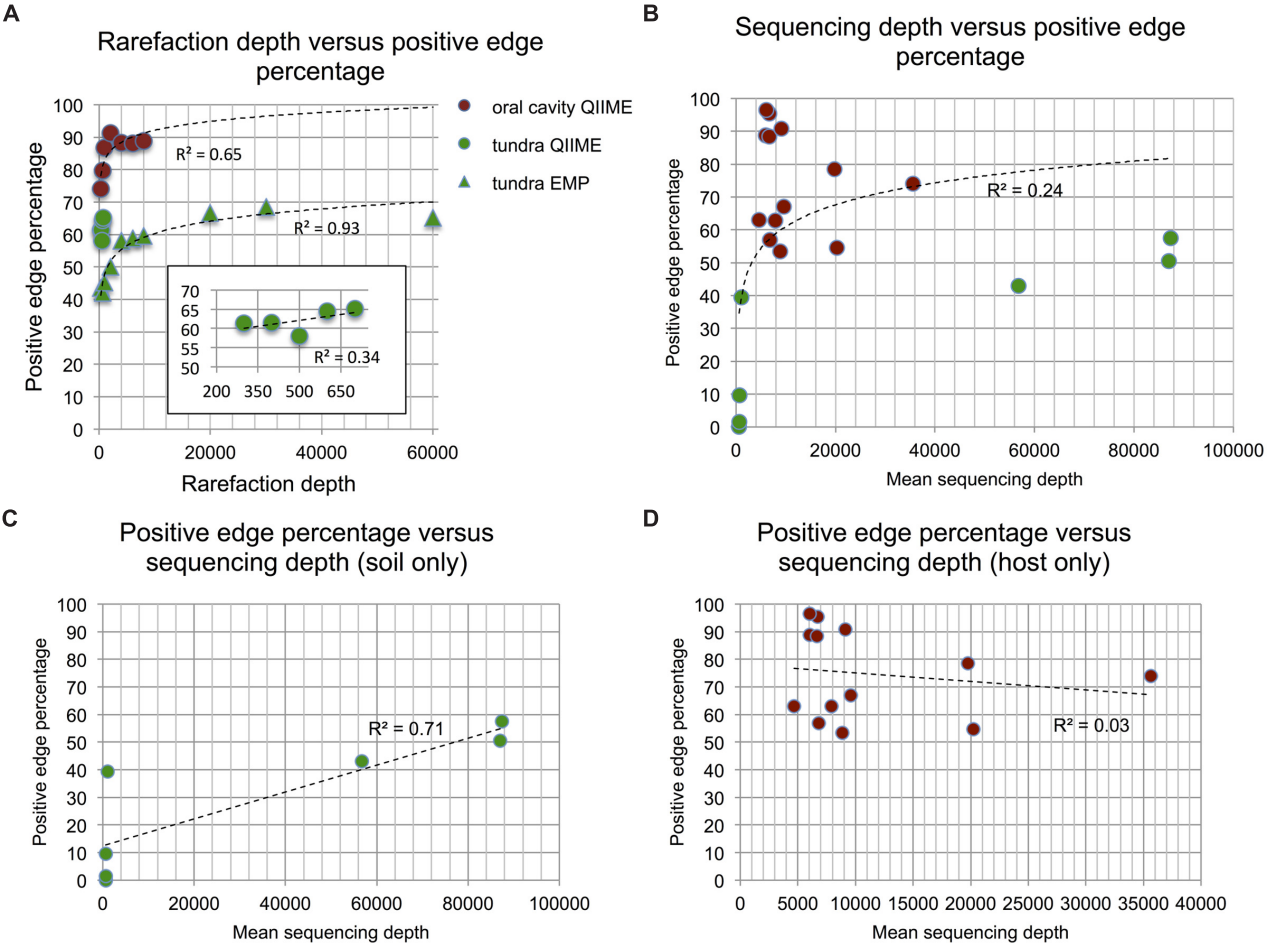
**Impact of sequencing depth.** Oral cavity and tundra networks were re-constructed from QIIME and EMP data rarefied to different depths (minimum occurrence was set to 13 for tundra QIIME, to 22 for tundra EMP and to 137 for oral cavity). In all cases, positive edge percentage (PEP) is correlated with sequencing depth (**A**; Spearman’s rho tundra QIIME: 0.7, *p*-value: 0.23, tundra EMP: 0.95, *p*-value: 2E-16, oral cavity: 0.75, *p*-value: 0.07). The trend line for oral cavity and tundra EMP is a logarithmic function of sequencing depth, whereas a linear trend line was fitted to tundra QIIME. Although sequencing depth is not significantly associated to PEP for all biomes (**B**; Spearman’s rho: 0.155, *p*-value: 0.51, logarithmic trend line), a significant correlation is detected when only soil biomes are considered (**C**; Spearman’s rho: 1, *p*-value: 0.0004). For host biomes, the correlation between PEP and sequencing depth is not significant (**D**; Spearman’s rho: 0.34, *p*-value: 0.26). Host data is colored in brown, soil data in green.

Another important difference between the selected host and soil datasets is sequencing depth, which averages to 723 reads per sample in QIIME soils (34,184 together with EMP soils) and to 22,428 reads per sample in QIIME host (11,386 together with HMP samples). Varying sequencing depth introduces biases, firstly because more taxa can be detected in more deeply sequenced samples and secondly because taxa co-vary with sequencing depth, resulting in spurious positive correlations. Without multiple-testing correction, the edge number increases with the taxon number (Supplementary Figure S3A). Assessment of significance and multiple-testing correction in simulations reduce this correlation, but do not entirely remove it. In agreement with the simulations, the edge number of the biome-specific networks is moderately correlated to taxon number (Supplementary Figure S3B). To investigate the second bias due to varying total counts, we simulated samples with different sequencing depths. The simulation confirms that varying sequencing depth increases PEP and that this bias is removed by either converting absolute into relative abundances (normalization) or by rarefying to the same sequencing depth (Supplementary Figure S4). Since we constructed networks from normalized matrices, we may not have sufficiently addressed the first bias, i.e. that taxon number increases with sequencing depth (though it is reduced by the removal of rare taxa). We therefore repeated network construction for rarefied data sets and found that PEPs of normalized and rarefied biomes were highly correlated (Spearman’s rho = 0.81, Supplementary Figure S5). We further explored the impact of sequencing depth by constructing networks from matrices rarefied to different depths and observed that PEPs of depth-specific networks increase non-linearly with rarefaction depth (**Figure 3A**). We then computed the correlation of biome-specific mean sequencing depth with PEP (**Figure 3B**), which was not significant in general, but was highly significant if only soil biomes were considered (**Figures 3B–D**). The taxon number as well as the mean of the all-versus-all Spearman distribution tends to increase with increasing sequencing depth (Supplementary Figure S6), which may account for the effect of sequencing depth on PEP. The increase of PEP with taxon number is also seen in our simulations (Supplementary Figure S9). However, although the EMP soils were sequenced more deeply than any of the host biomes considered in this study, their PEPs were below most of the host biome PEPs. We therefore conclude that despite the impact of sequencing depth on PEP, sequencing depth alone does not explain the difference between soil and host PEP.

### Evenness and Richness are Negatively Correlated to Positive Edge Percentage

**FIGURE 4:**
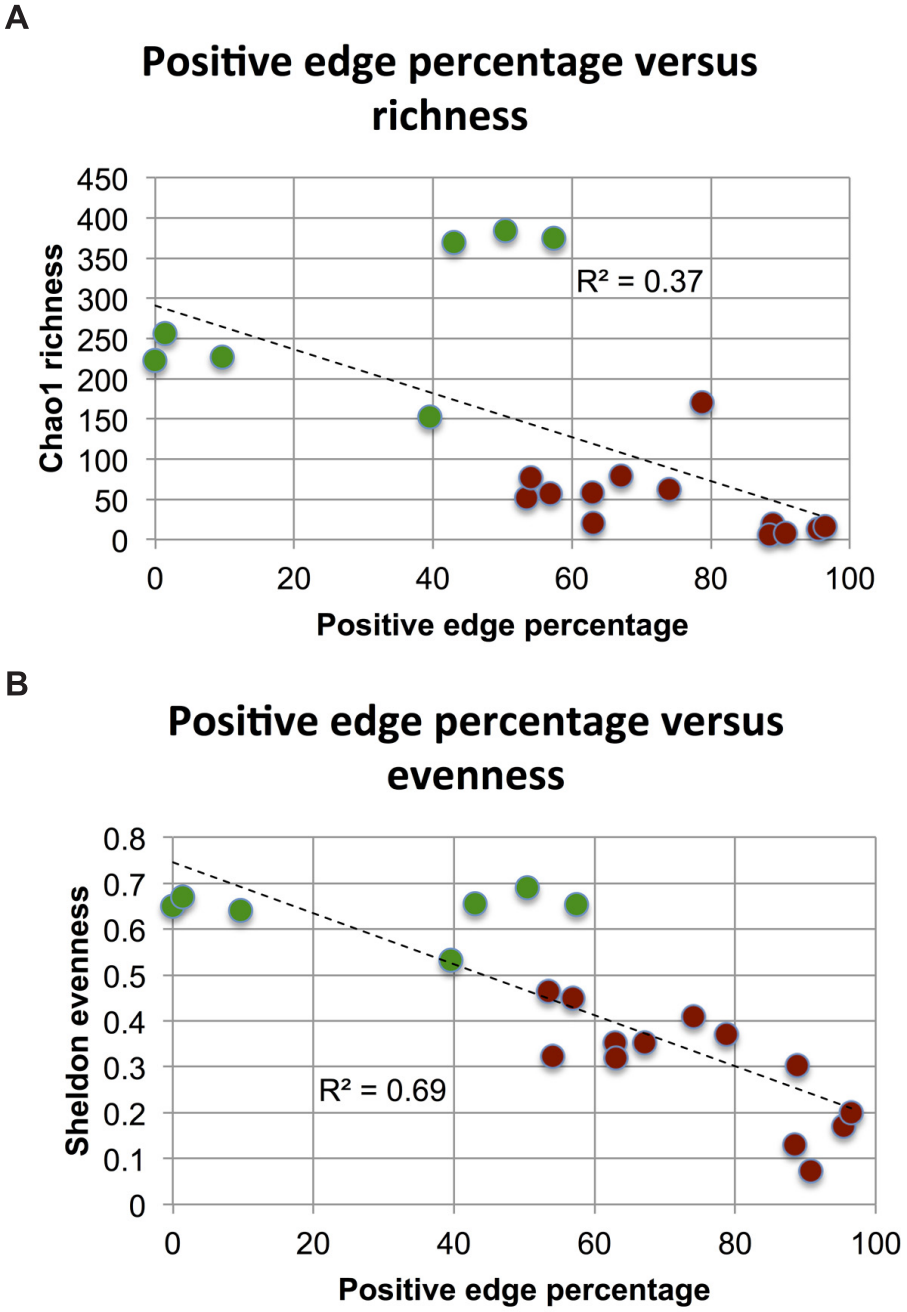
**Impact of richness and evenness.** When assessing richness (using median Chao1) and evenness (using median Sheldon’s index) in 20 (processed and rarefied) soil and host biomes, richness is found to be weakly anti-correlated **(A)** and evenness strongly anti-correlated to PEP **(B)**. Host data is colored in brown, soil data in green.

Next, we tested whether alpha or beta-diversity might drive the observed PEP difference. We did not find a significant difference for between-sample beta diversity of host and soil matrices (*p*-value of Wilcoxon rank sum test on Bray–Curtis distribution medians: 0.88) and therefore conclude that the differences between soil and host networks are not driven by sample heterogeneity. However, when assessing evenness (using Sheldon index), richness (with Chao1 estimator and OTU number) and alpha diversity (with Shannon index) on the biome matrices rarefied to the same sequencing depth (362 reads, to include as many samples as possible from the less deeply sequenced QIIME soils), we found soil matrices to be more even, rich and diverse than host matrices (Supplementary Figures S7 and S8), in agreement with previous results (Fierer and Lennon, 2011). Since diversity takes into account both evenness and richness, we computed the correlation of PEP to evenness as well as to richness to separate the effects of both and found that Chao1 richness as well as Sheldon evenness are both significantly anti-correlated to PEP (Spearman’s rho = –0.75 and –0.85, respectively, **Figures 4A,B**). It is known that diversity is sensitive to rarefaction depth (Lundin et al., 2012). Although Chao1 and Sheldon values did indeed vary with rarefaction depth, the ranking of biomes according to their evenness or richness was mostly preserved (Supplementary Figure S8).

### Impact of Taxon Number, Sample Number and Evenness in Simulations

We then explored whether simulated communities could reproduce the trends described above. In short, we generated count matrices with defined properties using the Dirichlet Multinomial distribution as in (Rosa et al., 2012; see Materials and Methods). The Dirichlet Multinomial does not model interactions between taxa and thus serves as a null model.

In count matrices simulated with the null model, PEP increased either with increasing taxon or decreasing sample number (with Spearman’s rho for median PEP of 1 and –1, respectively, Supplementary Figure S9). This contrasts with the observations in the biome-specific networks, where taxon and sample number are only moderately correlated to PEP (R2: 0.04 and 0.05, Spearman’s rho: –0.51 and 0.4, respectively). It has been noted recently that the Dirichlet Multinomial imposes negative correlations (Mandal et al., 2015), which explains the decrease in PEP with increasing sample size in the simulated matrices.

**FIGURE 5:**
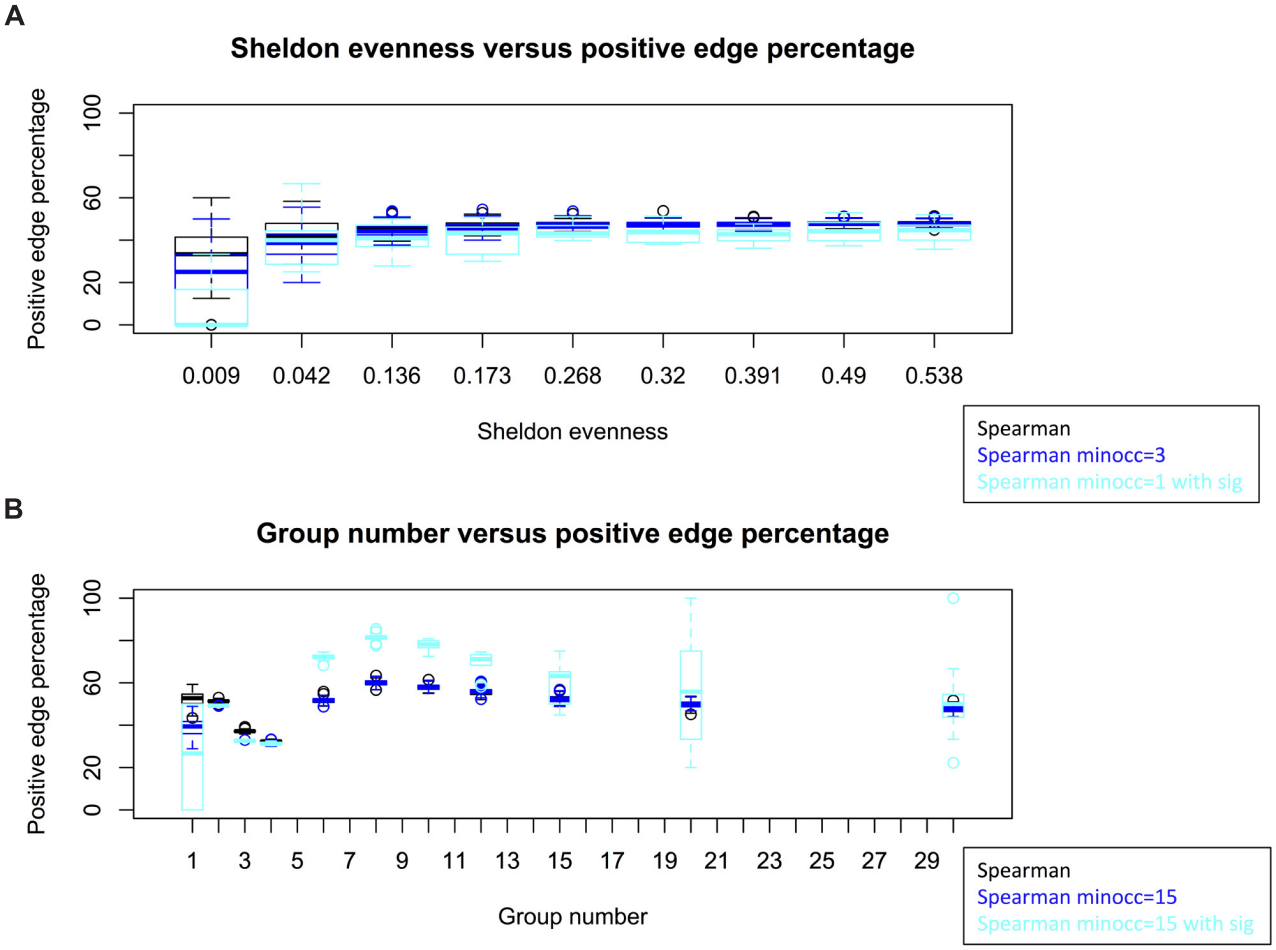
**Simulations with an interaction-free null model.** Evenness does not alter PEP in simulations, though the variance of PEP increases for low evenness, when most taxa are absent across all samples **(A)**. When introducing group structure, PEP varies non-linearly with group number **(B)**. Count matrices were simulated with 50 taxa and 10 samples **(A)** and 120 taxa and 60 samples **(B)** and networks were built using Spearman with cut-off at ±0.2. For the cyan box plots, significance was assessed by computing *p*-values from permutation and bootstrap distributions and correcting for multiple testing with Benjamini and Hochberg’s (1995) procedure. Matrix generation and network construction were repeated 100 times for each box plot (10 times when significance was assessed). Permutations and bootstraps were carried out with 100 iterations each. The parameter “minocc” refers to a filter step that removes all taxa occurring in less than the specified sample number.

Keeping taxon and sample number constant, we simulated count matrices of varying evenness. Within a large range of evenness, the average PEP does not change (**Figure 5A**). The variance of PEP increases for small evenness values, since fewer non-zero taxa are available for which correlations can be computed. Thus, the observed effect of evenness could not be reproduced with a model that does not account for taxon interactions.

We proceeded to investigate the effect of group structure. For this, we simulated a group as a set of taxa whose counts are much higher than the background across a sample sub-set and found a non-linear relationship between the number of simulated groups and PEP (**Figure 5B**). Thus, group structure could affect PEP.

### Less Prevalent Taxa in Soil Tend to Contribute More Negative Edges

**FIGURE 6:**
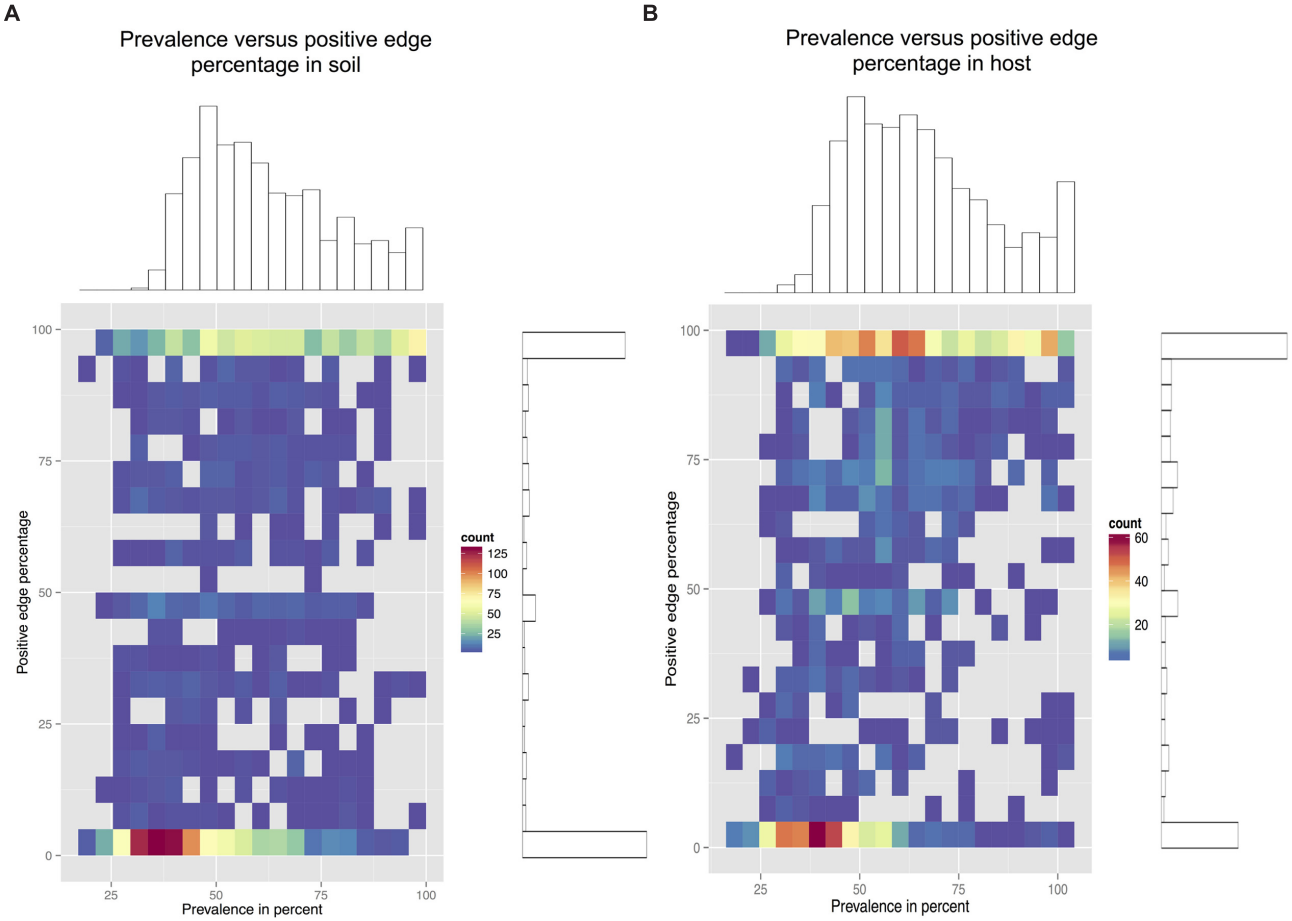
**Prevalence density plots.** The prevalence (measured as the percentage of occurrence across samples) and PEP in soil networks **(A)** and host networks **(B)** is divided in 20 bins and each node is placed in its bin combination. On the right and top of each density plot, the node-specific PEP and prevalence histograms are shown. In soil networks, node PEP tends to be low at lower prevalence, whereas in host networks, low PEP at low prevalence is balanced by high PEP at higher prevalence.

Since beta-diversity did not differ significantly between host and soil biomes, we looked at prevalence patterns instead. For this, we plotted soil and host node density for prevalence and PEP (**Figure 6**). Whereas the soil density plot has a single peak at low prevalence and PEP, the host density plot features as second peak at higher prevalence and PEP (Supplementary Figure S10 shows density plots for abundances).

**FIGURE 7:**
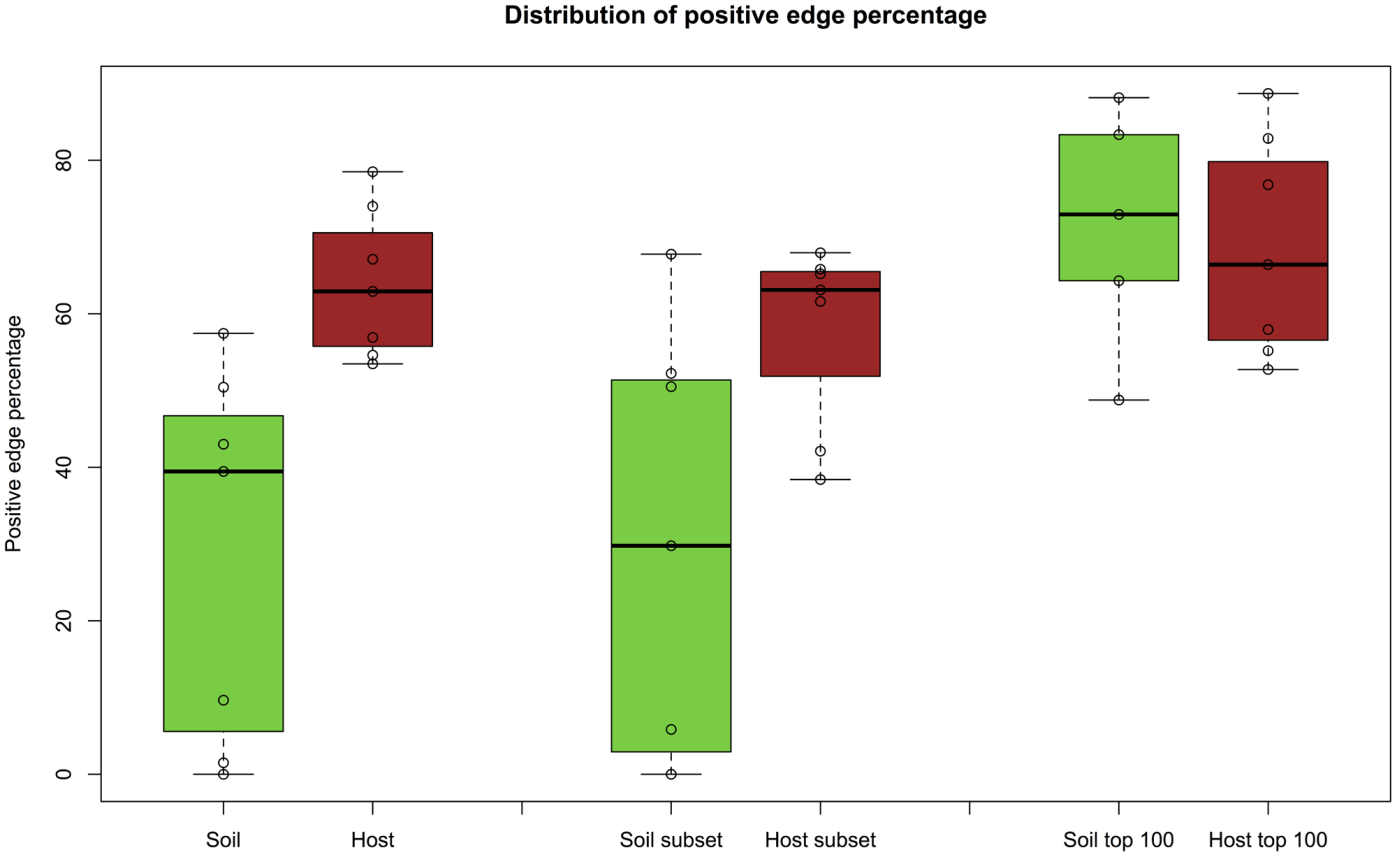
**PEP for top 100 prevalent taxa.** When networks are inferred from the top 100 prevalent taxa, the average PEP of soil networks increases, in contrast to host networks (fifth and sixth box plot). For comparison, PEP distributions of soil and host networks (first and second box plot) as well as soil and host networks excluding biomes with less than 100 OTUs (nasal cavity, skin, and vagina from the HMP dataset) and without higher-level taxa and metadata (third and fourth box plot) are also displayed. The Wilcoxon rank sum test for the latter case (third and fourth box plot) gives a *p*-value of 0.0014, whereas the PEP distribution difference for top-prevalent soil and host OTUs is no longer significant (*p*-value: 0.88).

To further investigate the impact of prevalence, we constructed networks from the top 100 most prevalent OTUs, i.e. from the 100 OTUs occurring in most samples. Equalizing row number across matrices also reduced their richness differences while preserving the differences in evenness. Whereas this selective removal of OTUs strongly increased the average PEP in soil, the change in average host PEP was minor (**Figure 7**), suggesting that less prevalent taxa in soil contribute to the difference in PEP between host and soil.

## Discussion

Here we show that microbial network inference can be applied in various contexts to study how environmental properties drive taxon associations (e.g. pH in the tundra network), to explore associations underlying community types (as for the enterotypes), or to identify novel potential ecological interactions (e.g. between Geobacteraceae and Nitrospirales). Furthermore, the simulations carried out to explore the impact of various matrix properties on PEP demonstrate the importance of data filtering, normalization and assessment of significance during network construction. If data are not filtered, rarefied or normalized or if significance is not assessed (e.g. when using Spearman correlation with arbitrary cutoffs), results may be biased by varying sequencing depth or may consist of a large number of false positives.

The significantly lower PEP of soil networks, in combination with the higher average clustering coefficient and network density of host networks, means that host microbial networks tend to be more interconnected and to contain more positive edges than soil networks. One can speculate that the higher PEP in host networks reflects a higher proportion of positive ecological interactions in host microbial communities (in the form of cross-feeding relationships, biofilms, etc.).

However, the soil-specific dependency of PEP on prevalence supports another hypothesis, which attributes the differences between soil and host to global community properties. When negative interactions tend to form predominantly between less prevalent community members, they are easier to detect in even than in uneven communities, since more sequencing effort is necessary in the uneven than in the even community to study the relationships between less prevalent members. This hypothesis explains the observed negative correlation between evenness and PEP for the biomes as well as the absence of this trend in the simulations (where neither negative nor positive interactions were introduced).

There may be other ways in which community structure impacts PEP; according to our simulations taxon group number may also play a role (**Figure 5B**). Taxon groups can be considered as the microbial equivalent to gene modules: the members of a taxon group respond together to varying environmental conditions and as a result are highly positively correlated, thus forming cliques. In environmentally driven taxon groups, the edges within and between groups can be considered as indirect, since group taxa co-vary mainly because of underlying environmental factors. The maximal possible number of negative between-group edges scales quadratically with the group number whereas the maximal number of positive within-group edges scales linearly. In agreement to this, the positive edge percentage decreased with larger numbers of simulated groups (**Figure 5B**).

When taxon groups include a large fraction of the taxa, they can be interpreted as alternative community types. Alternative community types can be the consequence of a direct or indirect disturbance or result from intrinsic system dynamics (Costello et al., 2009; Faust et al., 2015). While alternative communities have been identified in a number of body sites (Arumugam et al., 2011; Ravel et al., 2011; Ding and Schloss, 2014), the existence of soil community types has to our knowledge not yet been explored. In this context, the strongly significant difference in θ values, which are much higher in host communities, is of interest (Wilcoxon rank sum test *p*-value: 0.00003). Although sample heterogeneity as measured by the median sample-wise Bray–Curtis dissimilarity did not differ significantly between soil and host, its standard deviation was highly correlated with over-dispersion (Supplementary Figure S7). Based on these observations, we speculate that over-dispersion as well as the standard deviation of the Bray–Curtis dissimilarity may indicate the presence of alternative community types in a data set. Future comparative clustering analysis of biomes may shed further light on taxon groups, community types and their impact on positive edge percentage.

In addition, the connectivity patterns of taxa could reflect some underlying biases, such as a different depth of taxonomic resolution at the same sequencing similarity cut-off or varying degrees of cosmopolitanism. Cosmopolitanism, i.e. the wide-spread occurrence across different environments, has recently been linked to a tendency to form positive connections (Pascual-García et al., 2014). Although typical soil bacterial classes such as Acidobacteria, Chloracidobacteria and Solibacteres have lower aggregated PEPs and occur in fewer data sets than typical host-associated classes such as Clostridia and Bacilli (Supplementary Table S3), it is unclear whether this variation in class-specific PEP is driving the difference between soil and host communities or is in turn driven by it.

We also detected a weak positive and a moderate negative correlation of PEP with sequencing depth and richness, respectively. Sequencing depth and richness are weakly correlated to each other across all biomes (Spearman’s rho: 0.28), but highly correlated to each other and to PEP when only soil is considered (Spearman’s rho sequencing depth versus soil richness: 0.72, soil richness versus PEP: 0.72, soil sequencing depth versus PEP: 1). As expected, the effect of sequencing depth and consequently of richness is stronger in soil than in host-associated biomes, since at the same sequencing depth, more taxa (and taxon groups) will be discovered in an even than in an uneven community. However, when taking all biomes together, sequencing depth alone is not sufficient to explain the observed difference in PEP.

The elevated PEP in host-associated biomes can also be seen in the majority of the 18 host networks inferred from the HMP data by Friedman and Alm (2012), whereas the low soil PEP is in agreement with PEPs (averaging to 42%) reported in a recent study on 10 Brazilian soil sample sets (Lupatini et al., 2014). However, additional data sets and biomes need to be considered in future comparative network studies to validate the trends discussed here.

Overall, this study demonstrates the impact of global community structure properties on inferred microbial networks. This observation warrants thorough analysis of the whole range of community properties in microbial network inference, to avoid naive interpretations of these networks and flawed biological conclusions. Simulations such as those presented here will be instrumental in fully untangling the interplay between community structure and the interaction between its members.

## Conflict of Interest Statement

The authors declare that the research was conducted in the absence of any commercial or financial relationships that could be construed as a potential conflict of interest.

## Footnotes

1 http://environmentontology.org/

2 http://systemsbiology.vub.ac.be/conet
