# Supplementary material for: Cross-biome comparison of microbial association networks
Source: Front Microbiol. 2015 Oct 27;6:1200. doi: 10.3389/fmicb.2015.01200 (PMC4621437; doi:10.3389/fmicb.2015.01200)
Supplement: Supplementary file 1 [file Data_Sheet_1.DOCX]

***Supplementary Material***

**Cross-biome comparison of microbial association networks**

**Karoline Faust, Gipsi Lima-Mendez, Jean-Sébastien Lerat, Jarupon Fah**

**Sathirapongsasuti, Rob Knight, Curtis Huttenhower, Tom Lenaerts & Jeroen Raes^*^**

*** Correspondence:** Jeroen Raes: jeroen.raes@med.kuleuven.be

1. **Supplementary Data**

Additional supplementary material (including the Cytoscape file containing the inferred networks, network construction scripts and the R file) is available under the Biomes tab of the CoNet web site: http://systemsbiology.vub.ac.be/conet

1. **Supplementary Figures and Tables**

## Supplementary Figures

**A**

**B**

**Supplementary Figure 1. Network-wise average clustering coefficient and network density.** For each network, the average clustering coefficient **(A)** and the network density **(B)** are plotted for all, positive and negative edges. Network density is computed as 2E/N(N-1), where E is the edge number and N the number of taxa in the processed matrix.

**A**

**B**

**Supplementary Figure 2. Positive edge percentage (PEP) does not depend on sample size.** 10 different sub-sets of 50, 100 and 200 samples were selected from the oral cavity QIIME data set and networks were built for each sub-set. To quantify the variance due to the inference pipeline, network inference was also repeated 10 times for the full sample set (408 samples). As expected, variance increases with decreasing sample sub-set size **(A)**. For the tropical shrubland EMP dataset consisting of 85 samples, 40 of the samples were selected randomly 10 times and a network was constructed for each sample set **(B)**. The dashed line is the PEP obtained for the data sets originally.

**B**

**A**

**Supplementary Figure 3. Impact of taxon number in simulations and in biomes.** In simulations **(A)**, in the absence of filtering by significance, the edge number is increasing rapidly with taxon number (black boxes). Since the simulated data do not contain any interactions, all edges represent false positives. Discarding non-significant edges reduces the number of false positive edges, albeit not to zero. The computation of significance in our network construction pipeline (blue boxes, 100 iterations for the permutation and bootstrap distributions) reduces the number of false positives more strongly than a permutation test (green boxes). Count matrices were simulated with 50 samples and networks were built using Spearman with cut-off at +/-0.2. Matrix simulation and network inference was repeated 100 times to generate each black distribution and 10 times to generate each green and blue distribution. P-values were corrected for multiple testing with Benjamini-Hochberg's procedure (BH). In biome-specific networks **(B)**, total edge number correlates moderately with taxon number. Host data is colored in brown, whereas soil data is colored in green.

**Supplementary Figure 4. Impact of varying sequencing depth on positive edge percentage (PEP) in simulations.** Sequencing depth was either kept constant at 1,000 (first box plot) or varied from 500 to 1,000 (other box plots). Without normalization or rarefaction, varying sequencing depth strongly increases PEP (second box plot). Normalization (third box plot) or rarefaction (fourth box plot) remove this bias. Networks were built from simulated count matrices (with 50 taxa and 10 samples) using Spearman with cut-off at +/-0.2. Normalization was carried out by dividing each matrix entry by its column sum. Matrix simulation and network construction was repeated 100 times for each box plot.

**Supplementary Figure 5. Agreement of PEP for networks constructed from normalized and rarefied matrices.** The positive edge percentages of biome-specific networks constructed from normalized versus rarefied count matrices are highly correlated (Spearman's rho = 0.81, p-value: 1.8E-05). All matrices were rarefied to 600 counts per sample. To avoid taxa with zero occurrences after rarefaction, a minimal occurrence in at least 10 samples was imposed.

**Supplementary Figure 6. Impact of sequencing depth on the Spearman distribution.** The dependency of the positive edge percentage on the mean of the Spearman distribution computed for all OTU pairs is explored. Although there is only a weak correlation for all biomes put together (**A**; Spearman's rho: 0.49, p-value: 0.03), the correlation for soil is highly significant (**C**; Spearman's rho: 0.96, p-value: 0.003). **(B)** and **(D)** illustrate that the mean of the Spearman distribution tends to shift to the right with increasing sequencing depth in soil (**D**; Spearman's rho: 0.96, p-value: 0.003), whereas this tendency is much weaker for all biomes put together (**B**; Spearman's rho: 0.48, p-value: 0.035). In addition, **(B)** shows the strong dependency been sequencing depth and (scaled) OTU number.

**Supplementary Figure 7. Diversity of biomes.** Alpha diversity (measured with the Shannon index), richness (scaled Chao1 index) and evenness (Sheldon index) for the biome-specific count matrices rarefied to the same sequencing depth (362) is shown. Biomes are sorted by their median Shannon index. Beta diversity is assessed with sample-wise Bray-Curtis. The (scaled) over-dispersion parameter θ, which is obtained by fitting a Dirichlet-Multinomial distribution to each count matrix, is also shown. Each Chao1 index was divided by the maximum Chao1 index, whereas θ values were multiplied by 100. Except for θ, which was computed matrix-wise, each index was computed for each sample independently, thus each box represents the distribution of this index over the biome-specific matrix. Soil biomes have higher alpha-diversity, evenness and richness and lower over-dispersion than host-associated biomes. The low beta-diversity in the oral cavity of the QIIME dataset compared to the HMP data set is likely due to the different number of individuals sequenced (2 in the QIIME data set versus 242 in HMP). The standard deviation of the sample-wise Bray Curtis distribution is strongly correlated with the over-dispersion (Spearman's rho: 0.7, p-value: 0.0009).

**A**

**B**

**Supplementary Figure 8. Dependency of diversity measures on rarefaction depth.** Median Chao1 richness **(A)** and median Sheldon evenness **(B)** and are computed for biome-specific count matrices rarefied to different depths.

**Supplementary Figure 9. Positive edge percentage (PEP) of simulated count matrices is influenced by matrix dimensions.** Median PEP is perfectly correlated to taxon number (Spearman's rho: 1) and perfectly anti-correlated to sample number (Spearman's rho: -1) for any sample or taxon number, respectively. Networks were computed from simulated count matrices using Spearman with -/+ 0.2 cutoff. Matrix simulation and network inference was repeated 100 times for each box plot.

**Supplementary Figure 10. Abundance density plots.** The abundance (in quantiles) and positive edge percentage in soil networks **(A)** and host networks **(B)** is divided in 20 bins and each node is placed in its bin combination. On the right site of each density plot, the node-specific PEP histogram is shown.

## Supplementary Tables

**Supplementary Table 1. 16S rDNA sequencing information and count matrix properties.** Overview of the matrix properties of the biomes after preprocessing, including the number of rows with and without higher-level taxa. The table also lists the data source, sequencing platform and 16S region as well as the sample metadata used for network construction.

| **Biome (Source)** | **Contribu-ting studies** | **Sequencing platform and 16S region** | **Processed data: mean sequen-cing depth** | **Processed data: taxon number (without higher-level taxa) x sample number** | **Sample metadata** | | **Pro-cessed data: family number** |
| --- | --- | --- | --- | --- | --- | --- | --- |
| Soil biomes | | | | | | | |
| **Conife-rous forests (QIIME)** | 1 (NEON soils) | Roche FLX, V1V2 | 640 | 374 (310) x 90 | pH, water-content-soil | | 17 |
| **Grass-lands (QIIME)** | 1 (NEON soils) | Roche FLX, V1V2, | 655 | 423 (337) x 36 | pH, water-content-soil | | 31 |
| **Moist forests (QIIME)** | 1 (NEON soils) | Roche FLX, V1V2 | 692 | 427 (350) x 87 | pH, water-content-soil | | 18 |
| **Tundra (QIIME)** | 1 (Arctic soils, (Chu et al., 2010)) | Roche FLX, V1V2 | 1105 | 348 (288) x 33 | latitude, pH | | 16 |
| **Anthro-pogenic terrestrial (EMP)** | 1 | Illumina HiSeq, V4 | 87026 | 3032 (2512) x 70 | days since epoch, estimated elevation, estimated last sub-merged, elevation, hour, estimated years since sub plot, GPS elevation less estimated elevation, estimated years since submer-ged | | 118 |
| **Tropical shrub-land (EMP)** | 1 | Illumina HiSeq, V4 | 56797 | 2620 (2220) x 85 | depth, water-content-soil, eleva-tion, pH, annual season precipi-tation, latitude | | 87 |
| **Tundra (EMP)** | 1 (CryoCARB project) | Illumina MiSeq, V4 | 87439 | 2174 (1785) x 62 | elevation, pH | | 97 |
| Host biomes | | | | | | | |
| **Intestine (QIIME)** | 3 in human and 1 in humanized mice, (Turnbaugh et al., 2008;Costello et al., 2009;Turnbaugh et al., 2009;Caporaso et al., 2011) | Illumina, Roche FLX, V2, V4, V2V6 | 20234 | 314 (260) x 842 | elevation, latitude | 15 | |
| **Oral cavity (QIIME)** | 1 in human, (Caporaso et al., 2011) | Illumina, V4 | 35626 | 334 (230) x 408 | - | 28 | |
| **Skin (QIIME)** | 4 in human, (Fierer et al., 2008;Costello et al., 2009;Fierer et al., 2010;Caporaso et al., 2011) | Illumina, Roche FLX, V2, V4 | 19754 | 929 (713) x 1323 | elevation | 77 | |
| **Intestine 11BAY (HMP)** | 1 in human, (Methé et al.) | Roche FLX, V3V5 | 7913 | 247 (197) x 144 | - | 13 | |
| **Intestine 92WAU (HMP)** | 1 in human, (Methé et al.) | Roche FLX, V3V5 | 9621 | 322 (263) x 85 | - | 15 | |
| **Nasal cavity 11BAY (HMP)** | 1 in human, (Methé et al.) | Roche FLX, V3V5 | 6012 | 116 (62) x 149 | - | 17 | |
| **Nasal cavity 92WAU (HMP)** | 1 in human, (Methé et al.) | Roche FLX, V3V5 | 4678 | 124 (59) x 78 | - | 19 | |
| **Oral cavity 11BAY (HMP)** | 1 in human, (Methé et al.) | Roche FLX, V3V5 | 6798 | 221 (141) x 1521 | - | 23 | |
| **Oral cavity 92WAU** **(HMP)** | 1 in human, (Methé et al.) | Roche FLX, V3V5 | 8884 | 208 (126) x 771 | - | 24 | |
| **Skin 11BAY (HMP)** | 1 in human, (Methé et al.) | Roche FLX, V3V5 | 6687 | 109 (56) x 426 | - | 17 | |
| **Skin 92WAU (HMP)** | 1 in human, (Methé et al.) | Roche FLX, V3V5 | 6044 | 124 (55) x 305 | - | 23 | |
| **Vagina 11BAY (HMP)** | 1 in human, (Methé et al.) | Roche FLX, V3V5 | 6679 | 59 (28) x 195 | - | 7 | |
| **Vagina 92WAU (HMP)** | 1 in human, (Methé et al.) | Roche FLX, V3V5 | 9098 | 88 (40) x 110 | - | 12 | |

**Supplementary Table 2. Network properties.** In soil networks, the positive edge percentage (PEP) as well as the average clustering coefficient and the network density are significantly lower than in host networks, whereas the goodness of fit of the node degree distribution to a power law is significantly better in soil than in host.

| **Biome (Source)** | **Num-ber of nodes** | **Num-ber of edges** | **Positive edge percen-tage** | **Average clus-tering coeffi-cient** | **Average path length** | **Network density (with nodes instead of with taxa)** | **R2 of linear regression on node degree distribu-tion** |
| --- | --- | --- | --- | --- | --- | --- | --- |
| Soil biomes | | | | | | | |
| **Coni-ferous forests (QIIME)** | 20 | 12 | 0 | 0 | 1.2 | 1.72E-4 (0.06) | 0.96 |
| **Grass-lands (QIIME)** | 143 | 137 | 1.5 | 0 | 1.69 | 0.0015 (0.014) | 0.94 |
| **Moist forests (QIIME)** | 207 | 985 | 9.6 | 0.07 | 2.45 | 0.012 (0.05) | 0.84 |
| **Tundra (QIIME)** | 291 | 1954 | 39.6 | 0.38 | 2.41 | 0.03 (0.05) | 0.78 |
| **Anthro-pogenic terres-trial (EMP)** | 873 | 3128 | 50.4 | 0.23 | 2.91 | 4.6E-4 (0.008) | 0.86 |
| **Tropical shrub-land (EMP)** | 683 | 3496 | 42.68 | 0.25 | 2.86 | 5.44E-4 (0.015) | 0.81 |
| **Tundra (EMP)** | 743 | 2312 | 57.44 | 0.17 | 3.62 | 9.07E-4 (0.0084) | 0.92 |
| Host biomes | | | | | | | |
| **Intestine (QIIME)** | 221 | 2097 | 54.1 | 0.42 | 2.35 | 0.043 (0.086) | 0.65 |
| **Oral cavity (QIIME)** | 218 | 755 | 74 | 0.41 | 2.44 | 0.014 (0.032) | 0.76 |
| **Skin (QIIME)** | 565 | 2736 | 78.7 | 0.36 | 2.92 | 0.0064 (0.017) | 0.85 |
| **Intestine 11BAY (HMP)** | 163 | 275 | 62.91 | 0.21 | 2.07 | 0.009 (0.021) | 0.92 |
| **Intestine 92WAU (HMP)** | 241 | 704 | 67.05 | 0.26 | 2.64 | 0.013 (0.024) | 0.84 |
| **Nasal cavity 11BAY (HMP)** | 91 | 476 | 88.87 | 0.45 | 1.94 | 0.07 (0.116) | 0.24 |
| **Nasal cavity 92WAU (HMP)** | 102 | 649 | 63.02 | 0.3 | 2.04 | 0.085 (0.126) | 0.36 |
| **Oral cavity 11BAY (HMP)** | 216 | 2060 | 56.9 | 0.33 | 2.54 | 0.085 (0.089) | 0.39 |
| **Oral cavity 92WAU (HMP)** | 205 | 2153 | 53.47 | 0.32 | 2.47 | 0.1 (0.103) | 0.46 |
| **Skin 11BAY (HMP)** | 98 | 728 | 95.44 | 0.43 | 2.05 | 0.124 (0.15) | 0.27 |
| **Skin 92WAU (HMP)** | 95 | 645 | 96.47 | 0.39 | 1.86 | 0.085 (0.145) | 0.22 |
| **Vagina 11BAY (HMP)** | 54 | 283 | 88.45 | 0.44 | 1.64 | 0.165 (0.2) | 0 |
| **Vagina 92WAU (HMP)** | 83 | 550 | 90.76 | 0.37 | 2.16 | 0.144 (0.162) | 0 |

**Supplementary Table 3. Top 20 connected classes.** The degree and positive edge percentage summed over members of the top 20 connected classes across all networks is listed, as well as the number of occurrences across processed biome-specific matrices as a rough measure of cosmopolitanism.

| **Class** | **Aggregated degree** | **Aggregated positive edge percentage** | **Number of matrices where class is present** |
| --- | --- | --- | --- |
| **Clostridia** | 8071 | 71.95 | 16 |
| **Actinobacteria** | 6570 | 61.64 | 20 |
| **Bacilli** | 4174 | 67.68 | 18 |
| **Bacteroidia** | 3857 | 60.35 | 16 |
| **Alphaproteobacteria** | 3496 | 62.73 | 10 |
| **Betaproteobacteria** | 3294 | 53.92 | 20 |
| **Gammaproteobacteria** | 2899 | 60.75 | 19 |
| **Acidobacteria** | 1453 | 54.92 | 8 |
| **Solibacteres** | 1243 | 35.88 | 7 |
| **Fusobacteria** | 1194 | 72.78 | 9 |
| **Sphingobacteria** | 993 | 50.76 | 8 |
| **Flavobacteria** | 877 | 45.38 | 7 |
| **Deltaproteobacteria** | 753 | 48.61 | 9 |
| **Chloracidobacteria** | 670 | 31.34 | 3 |
| **Verrucomicrobiae** | 580 | 34.83 | 8 |
| **Spartobacteria** | 564 | 50.53 | 7 |
| **Spirochaetes** | 520 | 25.19 | 5 |
| **Erysipelotrichi** | 293 | 79.86 | 5 |
| **Planctomycea** | 270 | 48.52 | 6 |
| **Epsilonproteobacteria** | 258 | 82.95 | 4 |

**References**

Caporaso, J.G., Lauber, C.L., Costello, E.K., Berg-Lyons, D., Gonzalez, A., Stombaugh, J., Knights, D., Gajer, P., Ravel, J., Fierer, N., Gordon, J.I., and Knight, R. (2011). Moving pictures of the human microbiome. *Genome Biology* 12**,** R50. doi: 10.1186/gb-2011-12-5-r50.

Chu, H., Fierer, N., Lauber, C.L., Caporaso, J.G., Knight, R., and Grogan, P. (2010). Soil bacterial diversity in the Arctic is not fundamentally different from that found in other biomes. *Environmental Microbiology* 12**,** 2998-3006.

Costello, E.K., Lauber, C.L., Hamady, M., Fierer, N., Gordon, J.I., and Knight, R. (2009). Bacterial Community Variation in Human Body Habitats Across Space and Time. *Science* 326**,** 1694-1697. doi: 10.1126/science.1177486.

Fierer, N., Hamady, M., Lauber, C.L., and Knight, R. (2008). The influence of sex, handedness, and washing on the diversity of hand surface bacteria. *PNAS* 105**,** 17994-17999.

Fierer, N., Lauber, C.L., Zhou, N., Mcdonald, D., Costello, E.K., and Knight, R. (2010). Forensic identification using skin bacterial communities. *Proceedings of the National Academy of Sciences* 107**,** 6477-6481. doi: 10.1073/pnas.1000162107.

Methé, B.A., Nelson, K.E., Pop, M., Creasy, H.H., Giglio, M.G., Huttenhower, C., Gevers, D., Petrosino, J.F., Abubucker, S., Badger, J.H., Chinwalla, A.T., Earl, A.M., Fitzgerald, M.G., Fulton, R.S., Hallsworth-Pepin, K., Lobos, E.A., Madupu, R., Magrini, V., Martin, J.C., Mitreva, M., Muzny, D.M., Sodergren, E.J., Versalovic, J., Wollam, A.M., Worley, K.C., Wortman, J.R., Young, S.K., Zeng, Q., Aagaard, K.M., Abolude, O.O., Allen-Vercoe, E., Alm, E.J., Alvarado, L., Andersen, G.L., Anderson, S., Appelbaum, E., Arachchi, H.M., Armitage, G., Arze, C.A., Ayvaz, T., Baker, C.C., Begg, L., Belachew, T., Bhonagiri, V., Bihan, M., Blaser, M.J., Bloom, T., Bonazzi, V.R., Brooks, P., Buck, G.A., Buhay, C.J., Busam, D.A., Campbell, J.L., Canon, S.R., Cantarel, B.L., Chain, P.S., Chen, I.-M.A., Chen, L., Chhibba, S., Chu, K., Ciulla, D.M., Clemente, J.C., Clifton, S.W., Conlan, S., Crabtree, J., Cutting, M.A., Davidovics, N.J., Davis, C.C., Desantis, T.Z., Deal, C., Delehaunty, K.D., Dewhirst, F.E., Deych, E., Ding, Y., Dooling, D.J., Dugan, S.P., Michael Dunne, W., Scott Durkin, A., Edgar, R.C., Erlich, R.L., Farmer, C.N., Farrell, R.M., Faust, K., Feldgarden, M., Felix, V.M., Fisher, S., Fodor, A.A., Forney, L., Foster, L., Di Francesco, V., Friedman, J., Friedrich, D.C., Fronick, C.C., Fulton, L.L., Gao, H., Garcia, N., Giannoukos, G., Giblin, C., Giovanni, M.Y., Goldberg, J.M., et al. (2012). A framework for human microbiome research. *Nature* 486**,** 215-221. doi: 10.1038/nature11209.

Turnbaugh, P.J., Hamady, M., Yatsunenko, T., Cantarel, B.L., Duncan, A., Ley, R.E., Sogin, M.L., Jones, W.J., Roe, B.A., Affourtit, J.P., Egholm, M., Henrissat, B., Heath, A.C., Knight, R., and Gordon, J.I. (2008). A core gut microbiome in obese and lean twins. *Nature* 457**,** 480-484. doi: 10.1038/nature07540.

Turnbaugh, P.J., Ridaura, V.K., Faith, J.J., Rey, F.E., Knight, R., and Gordon, J.I. (2009). The Effect of Diet on the Human Gut Microbiome: A Metagenomic Analysis in Humanized Gnotobiotic Mice. *Science Translational Medicine* 1**,** 6ra14-16ra14. doi: 10.1126/scitranslmed.3000322.
